# A Phosphorus Burn

**Published:** 2015-03-05

**Authors:** Nigel Yong Boon Ng, Anas Abdullah, Stephen M. Milner

**Affiliations:** Johns Hopkins Burn Center, The Johns Hopkins University School of Medicine, Baltimore, Md

**Keywords:** phosphorus, burn, injury, management, grenade

**Figure 1:**
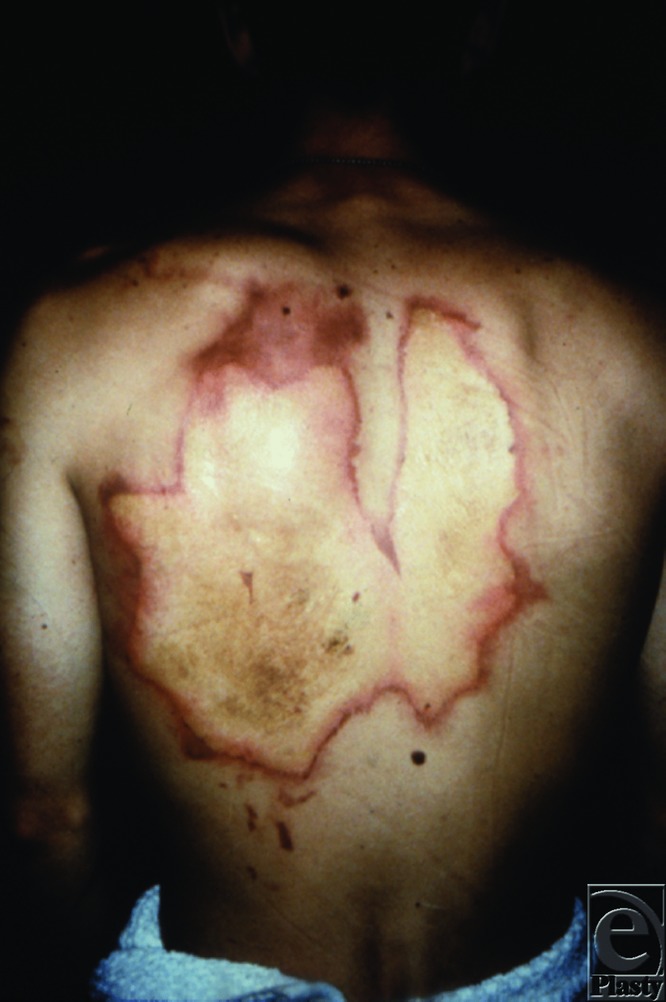


## DESCRIPTION

A 22-year-old soldier suffered a phosphorus burn to his back from the mishandling of hand grenades.

## QUESTIONS

1. **What are the common causes of phosphorus burns?**
2. **How does phosphorus burns cause injury?**
3. **What are the clinical features of phosphorus burns?**
4. **How are phosphorus burns managed?**

## DISCUSSION

These burns occur mainly in the military and munitions industry. Patients are usually injured by phosphorus-containing bombs and weapons in the field, or during factory production of munitions or fireworks. Barillo et al^1^ reported that phosphorus burns accounted for half the cases in 276 burnt patients treated in an American military burns center over 51 years. In homes, phosphorus is a component of common household products such as fertilizer and insecticide, and burns from these sources have been reported.

Phosphorus has a high tendency to stick to skin and when in contact, its highly lipid-soluble property can allow deepening of the burn injury into the subcutaneous tissue. Burning white phosphorus produces smoke, composed of mainly phosphorus pentoxide, an irritant to the eyes and mucous membrane of the respiratory tract. Systemic absorption from skin contact or inhalation causes erythrocyte hemolysis and multiple organ dysfunction.^2^^-^4

Phosphorus burns are typically severely painful, necrotic, and yellowish in color with a characteristic smell of garlic. They are commonly full-thickness burns, resulting from chemical and thermal insults. Systemic toxicity manifests in 3 phases: (1) In the first 8 hours, patient may experience gastrointestinal symptoms such as abdominal pain, nausea, vomiting, and diarrhoea; (2) From 8 hours to day 3, patient may be asymptomatic; (3) From day 4 to day 8, multiorgan failure and central nervous system dysfunction may result in death. Clinicians should also be wary of predictable complications such as hypocalcemia, hyperphosphatemia, and cardiac arrhythmia.^5^ Therefore, close monitoring of electrocardiogram and serum electrolytes are important.

There are no good-quality studies to recommend the optimal management strategy for phosphorus burns. However, there is consensus that thorough irrigation of the wound with saline or water and complete removal of phosphorus particles by debridement are pivotal. Identification of phosphorus particles is facilitated by use of either copper sulphate or ultraviolet light. Copper sulphate converts phosphorus to a black film of cupric phosphide, making small or embedded particles easily visible. However, its use is associated with renal failure and poor outcomes according to a recent Cochrane Review.^6^ Ultraviolet light provides a safer option and makes phosphorus particles glow. During the debridement process, burnt areas should be immersed in cold water to prevent phosphorus from igniting. While doing so, precautions should be taken to prevent hypothermia. The phosphorus removed should be placed in cold water for safe disposal. Hypocalcemia should be corrected with intravenous calcium gluconate (adult and pediatric dose: 0.1 to 0.2 mL/kg up to 10 mL/dose of a 10% solution). Unfortunately, there is no antidote for phosphorus toxicity.^7^ Therefore, complete debridement and supportive measures are the key management principles in this group of patients.
